# The DOMUS study protocol: a randomized clinical trial of accelerated transition from oncological treatment to specialized palliative care at home

**DOI:** 10.1186/1472-684X-13-44

**Published:** 2014-09-09

**Authors:** Mie Nordly, Kirstine Skov Benthien, Hans Von Der Maase, Christoffer Johansen, Marie Kruse, Helle Timm, Eva Soelberg Vadstrup, Geana Paula Kurita, Annika Berglind von Heymann-Horan, Per Sjøgren

**Affiliations:** 1 Department of Oncology, Rigshospitalet, Copenhagen University Hospital, Blegdamsvej 9, Copenhagen DK-2100, Denmark; 2 Department of Clinical Medicine, Faculty of Health and Medical Sciences, Copenhagen University, Copenhagen, Denmark; 3 Danish Cancer Society Research Center, Copenhagen, Denmark; 4 The Danish Institute for Local and Regional Research, Copenhagen, Denmark; 5 Danish Knowledge Centre for Palliative Care, Copenhagen, University of Southern Denmark, Copenhagen, Denmark; 6 Section of Palliative Medicine, Department of Oncology, Rigshospitalet, Copenhagen University Hospital, Copenhagen, Denmark; 7 Multidisciplinary Pain Centre, Department of Neuroanaesthesiology, Rigshospitalet, Copenhagen University Hospital, Copenhagen, Denmark

**Keywords:** Cancer, Home care services, Randomized controlled trial, Specialized palliative care, Palliative treatment, Patient care

## Abstract

**Background:**

The focus of Specialized Palliative Care (SPC) is to improve care for patients with incurable diseases and their families, which includes the opportunity to make their own choice of place of care and ultimately place of death.

The Danish Palliative Care Trial (DOMUS) aims to investigate whether an accelerated transition process from oncological treatment to continuing SPC at home for patients with incurable cancer results in more patients reaching their preferred place of care and death. The SPC in this trial is enriched with a manualized psychological intervention.

**Methods/Design:**

DOMUS is a controlled randomized clinical trial with a balanced parallel-group randomization (1:1). The planned sample size is 340 in- and outpatients treated at the Department of Oncology at Copenhagen University Hospital. Patients are randomly assigned either to: a) standard care plus SPC enriched with a standardized psychological intervention for patients and caregivers at home or b) standard care alone. Inclusion criteria are incurable cancer with no or limited antineoplastic treatment options.

**Discussion:**

Programs that facilitate transition from hospital treatment to SPC at home for patients with incurable cancer can be a powerful tool to improve patients’ quality of life and support family/caregivers during the disease trajectory. The present study offers a model for achieving optimal delivery of palliative care in the patient’s preferred place of care and attempt to clarify challenges.

**Trial registration:**

Clinicaltrials.gov Identifier: NCT01885637

## Background

The Capital Region of Denmark had in 2013 1,735,521 inhabitants with one of the highest incidences (0.6%) and prevalence (4.3%) of cancer in the world as well as a high cancer mortality rate (6.8%). Almost five thousand of the inhabitants of the Capital Region dies of cancer every year (0.25%) [1]. Thus, a substantial need for palliative cancer care is observed.

Studies have shown that most patients with advanced cancer prefer to spend the last part of their life at home (50-90%) [2,3]. However, in most western countries more than half of the patients with advanced cancer die in hospitals [3-9]. A recent systematic review by Gomes *et al.* showed that most people with different terminal diseases preferred to die at home and the majority of patients did not change preferences as their illnesses progressed [10]. Similar preferences were reported in a retrospective Danish survey, which indicated that the majority of cancer patients in the palliative care phase preferred to die in their own home (81%); however, the reported preference for dying at home decreased towards time of death (64%) [11]. Further, a recent prospective Danish study indicated that the majority (71%) of cancer patients in palliative care preferred to die in their own home [12]. According to the National Board of Health, 55% of Danish cancer patients died in hospitals, 18% in nursing homes or in specialist palliative care institutions, while only 26% died at home in 2005 [13]. A similar pattern is seen in other European countries, as the place of death in Europe is most frequently hospital or nursing home [14]. Even in the UK, which has a well-developed and comprehensive national palliative care system, a study analyzing all cancer deaths between 1993 and 2010 showed that 48% died in a hospital and 16% in hospices, while 25% died at home [15]. Timely and continuous involvement of General Practitioners (GP), nurses and - when necessary - a Specialized Palliative Care Team (SPT) seems to have a positive impact on the likelihood of dying at home [16,17]. Experience from the UK also showed that pronounced delay in the discharge from the hospital was a major obstacle to achieving the preferred place of palliative care and death [18].

In cancer care the availability of SPC seems to be a strong predictor for achieving home deaths [19-22]. A prospective study demonstrated that agreement and understanding between patients and informal caregivers about patients’ preferred place of palliative care seems to be a crucial prerequisite for obtaining death at home [23]. Grande and Ewing found that this consensus resulted in a substantial likelihood that the patient died at his or her preferred place [23]. It is interesting to note that place of death can be a proxy for place of care. A systematic review from 2006 explored the factors influencing death at home [24]. Among the main factors strongly associated with death at home, patient’s preference for home death and home care were clearly expressed [24].

Integration and coordination between oncological treatment, SPC and standard care may be the best way to organize the palliative care for patients with cancer [25]. This approach could give patients the advantage of timely access to care at home and simultaneous improvement of symptom control and quality of life [25]. Brumley *et al*. conducted a randomized controlled trial of palliative care at home with terminally ill patients (late-stage patients with cancer, chronic obstructive pulmonary disease, and congestive heart failure), which reported a significant increase in satisfaction with care 30 and 90 days after enrollment [26]. More patients died at home (71%) compared to those receiving standard care (51%) and the intervention group was less likely to visit emergency rooms or be admitted to hospitals (36% compared to 59% in the control group), resulting in significantly lower costs [26].

Another advantage of SPC at home is the possibility to support bereaved relatives and caregivers [27]. In most cases, the grief process is uncomplicated, however, for up to 15% of bereaved relatives grief may develop into a psycho-pathological condition [28]. The World Health Organization’s (WHO) definition of palliative care emphasizes that the informal caregivers’ health and needs should also be assessed and supported in palliative care [29]. Informal caregivers of patients with advanced cancer can experience deterioration of their own health and mental well-being [30]. Furthermore, caregivers’ wishes regarding the place of death for their loved ones and their perception of the social support they received seem to be significantly associated with place of death [31].

We also considered measures of telomere length in caregivers of cancer patients as a measure for an effect of the intervention. The pathophysiological consequences of caregiving have not been fully elucidated and several studies indicate that caregivers’ stress and strain may be associated with shorter telomere length, a marker of cellular aging. A study of 338 caregivers in the 2008–2010 Survey of the Health of Wisconsin observed significantly shorter telomere length associated with number of hours per week of caregiving, caring for a younger individual, and more strain. Caregivers with discordant levels of stress and strain (i.e., low perceived stress/high strain) compared with low stress/low strain had significantly shorter telomeres corresponding to approximately 10–15 additional years of aging [32].

Thus, the DOMUS study protocol will describe an investigation of accelerated transition from oncological treatment to SPC at home for patients with incurable cancer in order to support patients and their informal caregivers to reach and stay in their preferred place of care and death.

### Study objectives

The primary aim of DOMUS is to investigate whether an accelerated transition process from oncological treatment to SPC at home for patients with incurable cancer results in prolonged residence in their preferred place of care, which indirectly may increase the number of home deaths. The secondary aims are to investigate whether the intervention:

– relieves patients’ symptoms (pain, tiredness, drowsiness, nausea, vomiting, dyspnea, insomnia, appetite, constipation, and diarrhea)

– improves patients’ and the closest relatives’ quality of life (health related quality of life)

– relieves patients’ as well as caregivers’ psychosocial problems (depression, anxiety, dyadic coping, and caregiver burden)

– ameliorates the bereavement process among the closest relatives (anxiety, depression, loss- and restoration-oriented coping, prolonged grief, sleep, lifestyle)

– prolongs survival of patients

improves cooperation between professionals

– is cost-effective

## Methods/Design

### Study design

**Figure 1:**
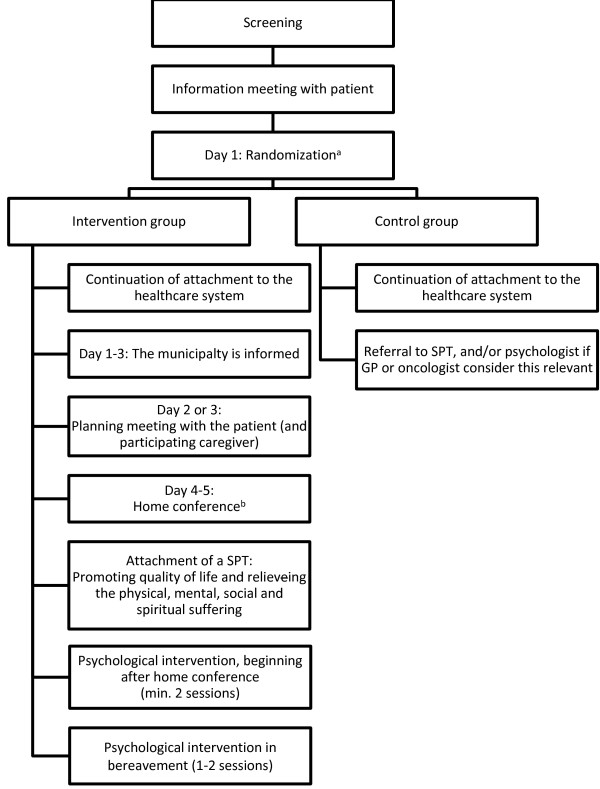
Description of the content of the intervention and the control group.

DOMUS is a controlled randomized clinical trial (RCT) with a balanced parallel-group randomization (1:1). Three-hundred-and-forty in- and outpatients in the Department of Oncology, Copenhagen University Hospital, will be randomly assigned to either an accelerated transition from oncological treatment to standard care plus continuing SPC enriched with a manualized psychological intervention at home (including nursing home) or standard care alone (Figure 1). The transition does not mean that oncological treatment has to be finalized.

### Patient selection

Inclusion and exclusion criteria are demonstrated in List of Inclusion and exclusion criteria of the DOMUS study:

### List of Inclusion and exclusion criteria of the DOMUS study

#### ***Inclusion criteria***

– Adult (at least 18 year old) cancer patients treated at the Department of Oncology, Copenhagen University Hospital

– Patients who want to spend as much time as possible in their own homes supported by a SPT

– Patients with incurable cancer

– Patients with no or limited antineoplastic treatment options^a^ or patients who resign antineoplastic treatment

– Patients living in the Capital Region, Denmark

– Written informed consent

a) Limited antineoplastic treatment options are specified in Table 1.

SPT = specialized palliative care team.

#### ***Exclusion criteria***

– Patients who have already been referred to a SPT

– Hospitalized patients who are not judged capable of being discharged home

– Patients who are admitted to other hospitals

– Patients who do not speak Danish well enough to answer the questionnaires

– Patients who are considered incapable of cooperating in the trial

#### ***Inclusion criteria for caregivers***

– Adults (at least 18 years of age)

– Written informed consent (in addition to that from the patient)

All included patients live in the Capital Region and are treated at the Department of Oncology, Copenhagen University Hospital. Limited antineoplastic treatment options listed in the inclusion criteria are dispelled in Table 1. A standard procedure is implemented to ensure that patients with varied diagnosis from the different clinics are screened evenly. In practice, it means that inpatients are screened on a daily basis and outpatients are screened following a sequential alternation procedure that guarantees an equal screening frequency of the six outpatient clinics involved in this study.

**Table 1:** Definition of limited antineoplastic treatment options

**Disease**	**Limited treatment options**
Breast cancer	Refractory to 3rd line antineoplastic treatment for metastatic disease
Lung cancer	Refractory to 1st line chemotherapy for metastatic/advanced disease
Gastrointestinal cancers	Refractory to 1st line chemotherapy for metastatic/advanced disease
Ovarian cancer/Uterine cancer	Refractory to 2nd line chemotherapy for metastatic/advanced disease
Cervical cancer/Vulva cancer	Refractory to 1st line chemotherapy for metastatic/advanced disease
CNS tumors	Refractory to concomitant/adjuvant chemotherapy
Prostate cancer/Bladder cancer/Penile cancer/Thyme cancer/Adrenal carcinomas	Refractory to 1st line chemotherapy for metastatic/advanced disease
Cancer of Unknown Primary origin (CUP)	Refractory to 1st line chemotherapy for metastatic/advanced disease
Head and neck cancer	Refractory to radiation therapy or surgery with curative intention

### Recruitment

Project nurses recruit patients from the oncology wards and outpatient clinics. From June 2013 to June 2015 three project nurses daily screen in- and outpatients from the Department of Oncology, Copenhagen University Hospital, and contact selected patients after having examined medical records to determine whether the patients are eligible for inclusion in the trial.

Before the patient agrees to participate in DOMUS, an information meeting will be held, to give the patients and closest relative oral and written information about the trial. If the patient gives consent to participate, an informal caregiver, appointed by the patient, is also invited to participate. Upon consent, the baseline questionnaires will be completed followed by randomization of the patient. A blood sample will be taken from the caregiver within a week to measure the leukocyte telomere length (a stress level marker).

### Randomization

The randomization is computer-generated by a statistician who is not attached to the project, the allocation sequence and block size are unknown to the involved nurses, who recruit patients and collect data. The allocation ratio will be 1:1. After the patient have given informed consent and filled out the baseline questionnaire, the nurses draw a sealed envelope, which is packed by uninvolved staff and contains information about randomization group.

### Intervention group

In the intervention group several steps will take place. A meeting is held with a research nurse one or two days after randomization, where the patient (and informal caregiver) discusses the patient’s wishes for treatment and care at home and how these can be reconciled with the caregiver’s wishes. The patient and caregiver’s perceptions of challenges and concerns related to home care are explored and addressed. Shortly after the meeting, any necessary changes regarding the home interior are planned.Four to five days after randomization a home conference is held including the patient, caregiver, representatives of the SPT, a district nurse, and if possible the GP and project psychologist. The SPT, in collaboration with the GP and the district nurse, is now responsible for the distribution of tasks related to further treatment and care (Figure 1).

Only two of nine SPTs in the Capital Region have psychologists to support and treat patients and their relatives in their homes [33]. Therefore, in order to support patients and caregivers included in the intervention group, a standardized psychological intervention along with the regular SPT intervention (physician and nurse) is added. Patients and caregivers receive a psychological supportive care intervention, beginning after the home conference. The intervention is conducted based on a manual, developed for the DOMUS trial to describe and standardize the use of a joint patient-caregiver psychological intervention in a palliative care setting.

### Control group

In the control group, the patients follow current practice in the healthcare system, being standard care as described below. If the patient is allocated to the control group, an additional meeting with the patient and the informal caregiver will be offered to clarify the options available in case of unmet palliative needs. A patient allocated to the control group can obtain equivalent elements of treatment and care as a patient allocated to the intervention group; however, the flow and sequence may be different.

### Standard care

Patients allocated to both groups will remain patients of the Department of Oncology at Rigshospitalet and continue oncological treatment if the oncologist find it relevant. General care provided according to the precepts of the Danish healthcare system includes in-hospital treatment as well as a GP, a GP out-of-hours service and access to 24-hour home nursing or nursing homes. Patients and caregivers may – if needed - receive psychological counseling through referral from their GP. Patients can be referred to SPC if their GP and/or oncologist judge this to be relevant. The current practice is referral due to complex palliative care needs.

The Danish healthcare system is financed through taxes and is free of charge at the point of receipt.

### Specialized palliative care

The definition of SPC in Denmark is based on the European Associations of Palliative Care’ statement [14]: “Specialist palliative care services require a team approach, combining a multi-professional team with an interdisciplinary mode of work. Team members must be highly qualified and should have their main focus of work in palliative care” [14]. At the moment 18 hospices, four hospital wards and 28 SPTs fulfill these requirements throughout the country. In the Capital Region there are nine SPTs; four of them are hospice-based and five are hospital-based [33].

### Assessments

Project nurses collect baseline data from patient records regarding the medical history of the included patients. Patients and caregivers are asked to complete questionnaires (Table 2) before they are randomized and after 2, 4, and 8 weeks and 6 months. Caregivers (bereaved) will be asked to fill in questionnaires (Table 2) two weeks and 2, 7, 13 and 19 months after the patient’s death. The selection of questionnaires was based on previous experience, validation and availability in the Danish language. As we do not want to burden the participants it is stated in each of the questionnaires that patient/caregiver/bereaved are welcome to fill it in over two days. Each questionnaire package is estimated to take approximately 30 minutes to fill in.

**Table 2:** Assessments in The DOMUS study

	**Investigators**	**Patient**	**Caregiver**	**Bereaved**^**a**^
Medical History	• Cancer Diagnosis, Co-morbidity, Time of Cancer Diagnosis, Current Disease Stage, ECOG Performance Status etc.	Baseline		
Reported Outcomes (questionnaires)	• Relationship Status, Children, Education	Baseline	Baseline	
	• Wishes for place of care and treatment	Baseline	Baseline	
		Week 2,4,8	Week 2,4,8	
		6 months	6 months	
	• Quality of life questionnaire – Core (EORTC-QLQ-C30) [34]	Baseline		
		Week 2,4,8		
		6 months		
	• Edmonton Symptom Assessment System (revised version) (ESAS-r) [35]	Baseline		
		Week 2,4,8		
		6 months		
	• Hospital Anxiety and Depression Scale (HADS) [36]	Baseline		
		Week 2,4,8		
		6 months		
	• Symptoms priority	Baseline		
		Week 2,4,8		
		6 months		
	• Symptom Checklist-92, anxiety and depression subscales (SCL-92) [37]	Baseline	Baseline	Week 2
		Week 2,4,8	Week 2,4,8	2, 7, 13, 19 months
		6 months	6 months	
	• Modified Medical Outcomes Study Social Support Survey (mMOS-SS)^b^[38]	Baseline	Baseline	Week 2
				2, 7, 13, 19 months
	• Dyadic Coping Inventory (selected subscales) (DCI)^b^[39]	Baseline	Baseline	
		Week 2,4,8	Week 2,4,8	
		6 months	6 months	
	• Relationship ladder^b^[40]	Baseline	Baseline	
		Week 2,4,8		
		6 months		
	• Zarit Burden Interview (ZBI) [41]		Baseline	
			Week 2,4,8	
			6 months	
	• Experiences in Close Relationships-Short Form (ECR) [42]		Baseline	7, 13, 19 months
	• Medical Outcomes Study Short Form (SF-36) [43]		Baseline	Week 2
			Week 2,4,8	2, 7, 13, 19 months
			6 months	
	• Inventory of Daily Widowed Life (IDWL) [44]			Week 2
				2, 7, 13, 19 months
	• Pittsburgh Sleep Quality Index (PSQI) [45]			Week 2
				2, 7, 13, 19 months
	• Prolonged Grief Disorder (PG – 13) [46]			7, 13, 19 months
	• Lifestyle single items			Week 2
				2, 7, 13, 19 months
Blood samples	• Telomere length		Baseline	2 months
Databases^c^	• Number of hospital admissions	From Baseline to 6 months	From Baseline to 6 months	
	• Number of inpatient bed days			
	• Number of outpatient visits to hospitals			
	• Number of visits to GP			
	• Number of visits to the emergency room			
	• Number of visits to the emergency physician			
	• Number of visits to specialists			
	• Treatments in public hospitals			
	• Prescribed drugs			
	• Place of care and death			
Healthcare services	• Health insurance charges	From Baseline to 6 months	From Baseline to 6 months	
	• Pharmacy prices			

^a)^The bereaved is the primary caregiver, ^b)^Only patients with participating relatives, ^c)^The National Patient Register, Health Insurance Registry, Medicine Databases.

In addition, patient’s informal caregivers are asked for a blood sample.

Databases will be used to collect data about the patients and caregivers use of healthcare (Table 2). All planned assessments, questionnaires, and medical records are shown in Table 2.

### Statistical methods

#### ***Sample size estimations***

Sample size was calculated considering the primary purpose of this trial, which is to prolong patients’ residence at home and to increase the number of patients who die at home by 15 - 20%. The latter purpose serves as a proxy for the first purpose. With a power of at least 80%, and an effect difference of 15% at a significance level of 0.05, 170 patients are required in each study arm (Ficher´s exact test). On the basis of this and an expected dropout rate of 10-15%, we plan to randomize 380 patients for the study.

### Statistics analyses

Microsoft Access 2010^®^ will be used as a database of information on included patients. Data from the questionnaires are scanned via ReadSoft Forms© (ReadSoft AB, Helsingborg, Sweden) into another database. Before data analyses the two databases will be merged.

The data analyses will comprise four main procedures:

1. Longitudinal analyses by multivariate linear mixed effect regression model, in which changes over a period of 2, 4 and 8 weeks will be compared. This will include analysis of a) whether there is a significant difference in the intervention and control group during the follow-up, and b) if the changes are similar for the two groups in between the measurements at respectively 2, 4 and 8 - week follow-up. In order to determine whether there is a difference in survival between the two groups Kaplan-Meier plot is used. Survival in the two groups is also compared in a Cox regression, which can be controlled for the same variables as the analyses of questionnaire data.

2. Intention to treat analyses, in which changes in outcome variables between the intervention and control group of caregivers, will be tested by linear “mixed-effect” regression models for repeated measurements. These analyses will be conducted for outcomes both before and after the patient’s death. To investigate the possible effect modifications, relevant interactions will be included, among these interactions between the randomization group and time since baseline, time since diagnosis, and baseline values of outcomes.

3. Data collected in semi-structured interviews after the intervention will be analyzed qualitatively to identify how a selection of the bereaved caregivers have experienced the care of the patient in their homes and how they have experienced the intervention.

4. Cost-effectiveness analyses, in which multivariate regression models will be used to analyze differences in costs between the intervention- and control-group, and to relate these to observed differences in effects.

5. Data collected in 10 – 12 focus groups will be analyzed to describe the experiences of the professionals involved in the trial; their evaluation of cooperation and quality of treatment and care in the patient trajectories.

### Trial plan

The trial is completed when the 340 patients have been randomized and after the last patient has been followed six months from baseline. Data collection is estimated to go on for 2 years. However, the caregivers will continue to be followed 19 months after the death of the patients.

### Ethics

The National Committee on Health Research Ethics, Denmark (37237) and The Danish Data Protection Agency (2007-58-0015) have approved the DOMUS trial.

The DOMUS trial has the logical consequence that the two groups of patients do not receive the same care. This can be justified mainly by the lack of evidence of whether accelerated transition from oncological treatment to SPC at home as a whole has any effect on place of care and death, symptoms/problems, quality of life, survival and caregivers’ health and grief. Further, it should be emphasized that the patients in the control group will receive the same treatment and care as they previously had, and as others in similar circumstances. As already mentioned the control group has the possibility of being referred to the same treatment and care as the intervention group with the exception of the psychological intervention. However, the GPs can refer patients in the control group to psychological treatment outside of the trial. Finally, the study staff will not prevent a potential crossover of patients.

## Discussion

The DOMUS trial is a comprehensive RCT in incurable cancer patients – to our knowledge the first of its kind - including outcomes from the individual patient and closest relative, as well as healthcare research of economy and organization. The primary goal of this trial is to ensure accelerated transition from hospital to SPC at home, so patients with incurable cancer can obtain timely transferal to and prolonged residence in their own homes [11]. The model is based on already existing resources, which are organized in a fixed sequence – a “fast-track lane” - with the addition of a manualized psychological intervention. The psychological intervention aims to support patients and caregivers jointly, as coping with severe illnesses depends, not only on the individual, but the dyad [47]. The effect of accelerated transition to a SPT enriched with a psychologist intervention will hopefully provide further knowledge of the needs of patients and caregivers as well as the costs and the expenses associated with the intervention. Finally, our study will, due to the transparency (Ref: Clinicaltrials.gov Identifier: NCT01885637) and the present paucity of comprehensive RCTs regarding clinical, organizational and health economical aspects of SPC, be valuable for decision-making in our region as well as in countries with comparable healthcare systems, and hopefully also be a source of inspiration for other healthcare systems [31,48]. In developing the DOMUS trial, the authors have discussed several issues, which may raise criticism and pose challenges to feasibility and influence outcomes. One of them is obtaining agreement between patients and relatives about place of care. In our population, we have no data on the degree of agreement, which of course may be highly dependent on the quality and extent of information. In the DOMUS trial, this information is given by trained research nurses and based on an information manual regarding SPC at home. In a retrospective study, 80% of the patients and relatives agreed on the preferred place of death, and 20% of the relatives changed their minds (in 51% of these cases the preference moved from home to another setting) [49]. However, in this study the patients’ preferred place of death was given by the relatives after the deaths of their loved ones, and therefore the “true” proportion of agreement is uncertain [49]. Based on the retrospective data and our clinical experience, we anticipate that thorough and proper information about SPC at home will create agreement in the majority of cases.

Another issue of discussion in the DOMUS trial has been to define the inclusion criteria. Cancer patients who are eligible for SPT can be defined in many ways depending on the purpose of the study or clinical outcomes. A RCT by Temel *et al.* demonstrated that patients with newly diagnosed metastatic lung cancer benefitted from referral to a SPT, as these patients achieved better quality of life, were less depressed and lived longer than patients in a control group who received standard therapy [48]. Thus, the Temel study was engaged in early intervention of SPC for a specific cancer diagnosis [48], whereas our study is engaged in defining patients with different cancer diagnoses treated at a comprehensive cancer center in need of SPC at home. Therefore, in addition to the expressed wish (and agreement between patient and closest relative) to be cared for at home, we have selected the inclusion criteria “incurable cancer with limited or no antineoplastic treatment left” (List of Inclusion and exclusion criteria of the DOMUS study). Further, we have characterized the meaning of “incurable cancer with limited or no antineoplastic treatment left” for all involved diagnoses (Table 1). Hence, we have sought to define a palliative patient population suitable for and in need of SPC at home, which of course can be adapted to other diagnoses as well. By defining limited treatment options, we have established a set of criteria that are already an integrated part of oncological treatment. With these criteria, we may in the future enable clinicians to proactively identify patients in the oncological treatment trajectory, who may benefit from SPC. In the initial phase of the inclusion of patients ECOG performance status 2–4 was an inclusion criterion. However, after a year of inclusion only 66 patients were enrolled. In order to speed up recruitment of patients as well as attempting to study earlier SPC [29,48] we have decided to remove the criteria (ECOG performance status 2–4). Further, by deleting the inclusion criteria it will be possible to investigate the difference effect of SPC in low and high ECOG performance status patients. The changes will be reported to clinicaltrial.gov.

The planned intervention is complex, including several sequential elements, and therefore it will not be possible to evaluate single elements of this multimodal trajectory intervention. The accelerated transition from oncological treatment requires a flexible primary care setting and a SPT ready to take care of the patient at home within five days of enrolment in the trial. The SPTs in the Capital Region of Denmark have their own individual referral guidelines and operate in less well-defined local areas. To ensure an equal distribution of patients to the different SPTs, the DOMUS protocol allocates patients to relevant SPTs according to uniformed referral guidelines and geographical based allocation. The geographical distribution of adequate SPC and the collaboration between SPTs, GPs, and district nurses is a rather simple prerequisite for the delivery of good palliative care. In addition, this issue may play a significant role even in countries with well-developed palliative care. In the UK with a comprehensive coverage of palliative care obtainable across the country, there seems to remain a lot of inequity, including that relating to geography, diagnosis, age and ethnicity [50]. In the DOMUS trial we will, in addition to the introduction of uniformed referral guidelines and geographical based allocation, further investigate these issues by adding qualitative interviews with patients, informal caregivers and healthcare professionals.

Currently a psychological intervention is not included in the standard SPT in Denmark, although it is recommended that a SPT consist of doctors, nurses, and one or more other medical professionals [14,51]. In five out of nine SPTs in the Capital Region a psychologist is already part of the hospice/palliative care unit team, but as the capacity is limited the psychologists are only used for in-patients except in two palliative care units, where they can participate in SPC at home [32]. However, as all SPTs of the region have agreed to participate in the DOMUS trial, the standardized manualized psychologist intervention is applied to all patients and relatives allocated to the intervention group. Thus, if the multimodal trajectory intervention enriched with a targeted psychologist intervention proves to be successful it may inspire the future model for SPC.

The intervention proposed in the DOMUS trial requires a consistent and intensified collaboration between healthcare professionals in the hospital, the primary healthcare sector and the SPTs in order to give the patients the best possible care at home. The DOMUS study is primarily important, because it can show whether the intervention can improve the treatment of palliative patients and provide a model for how the SPC should be organized in the community. It is essential to find out whether place of care and death, longer survival and healthcare benefits are associated with the intervention. As the organizational and financial consequences of the model will also be investigated, there is a direct basis on which decisions can be taken regarding the organization of future palliative care. In addition to national importance of the study it has great international relevance, since high quality evidence from RCTs is sparse in palliative care, and no RCTs with an accelerated transition from oncological treatment to continuing SPC at home in patients with incurable cancer have previously been published.

## Competing interests

The authors declare that they have no competing interests.

## Authors’ contributions

MN, HM, CJ, MK, HT, AH, and PS designed the study and developed the methods; KB, GK, and EV helped to improve the methods. MN, KB, AH, HM, CJ and PS conduct the practical daily activities of the trial. HT, CJ, HM, and PS supervise the study development. PS, CJ, HT, GK, EV supervise the PhDs students and their activities in the study (MN, KB, and AH). MN, HT, CJ, AH and PS drafted the manuscript. All authors read, made suggestions, revised, and approved the final manuscript.

## Pre-publication history

The pre-publication history for this paper can be accessed here:

http://www.biomedcentral.com/1472-684X/13/44/prepub
